# Research on the Physical Properties of an Eco-Friendly Layered Geopolymer Composite

**DOI:** 10.3390/ma17194937

**Published:** 2024-10-09

**Authors:** Agnieszka Przybek, Michał Łach

**Affiliations:** Faculty of Material Engineering and Physics, Cracow University of Technology, Jana Pawła II 37, 31-864 Cracow, Poland; michal.lach@pk.edu.pl

**Keywords:** geopolymer concrete, natural fiber, composite reinforcement, alkaline activation, modern building material, CO_2_ reduction

## Abstract

Building envelopes with natural fibers are the future of sustainable construction, combining ecology and energy efficiency. The geopolymer building envelope was reinforced with innovative composite bars and two types of natural insulation (coconut mats and flax/hemp non-woven fabrics) were used as the core material. A 10 mol sodium hydroxide solution with an aqueous sodium silicate solution was used for the alkaline activation of the geopolymers. The purpose of this study was to confirm the feasibility of producing geopolymer composites with insulating layers made of renewable materials, which would have compressive strengths like those of C25/30-grade concrete and thermal conductivity coefficients like those of lightweight concrete. This publication presents the results of physicochemical tests on the base materials (oxide (XRF) and mineral phase (XRD) analysis as well as morphology and EDS) and studies the physical (density measurements), mechanical (flexural and compressive strength tests) and insulating properties (thermal conductivity measurements) of the finished sandwich partitions. The composites achieved a flexural strength of 7 MPa, a compressive strength of up to 30 MPa and a decrease in the thermal conductivity coefficient of about 60%. The research demonstrates contribution to sustainable construction by developing geopolymer composites, offering both structural integrity and superior thermal insulation. This innovation not only reduces reliance on traditional, carbon-intensive materials but also promotes the use of eco-friendly resources, significantly lowering the carbon footprint of construction. The integration of natural fibers into geopolymer matrices addresses key environmental concerns, advancing a rapidly growing field that aligns with global efforts toward energy efficiency, waste reduction, and circular economy principles in building design.

## 1. Introduction

Building partitions with natural fibers are becoming increasingly popular due to their eco-friendly properties, good insulation and low CO_2_ emissions. Natural fibers such as flax, hemp, jute, cotton, natural fiber wool, bamboo or coconut fibers are used to produce a variety of building materials that can be used in wall partitions, roofs, floors and as insulation [1,2,3,4,5,6,7,8]. Building envelopes with natural fibers are the future of sustainable construction, combining ecology, health and energy efficiency [9,10,11,12].

Composite materials are one sector of the modern building envelope. The main characteristics of these materials include low density, high stiffness and very good strength. One group of materials in which natural fibers are often used is polymers [13,14,15,16,17,18,19,20]. Currently, there is a growing demand for alternative construction technologies that aim to reduce construction waste, manage materials perceived as by-products of various technological processes and reduce energy consumption in the life cycle of buildings. Partitions are one of the most important parts of any building project, as, without partitions, the building plan cannot be structural or decorative [21,22]. Many materials are available on the market for the production of partition walls, including OSB [23,24], regips [25,26], plasterboard [27,28], chipboard [29,30], gypsum-fiber board [31,32], cement-fiber board [33,34], furniture board [35,36] and gypsum blocks [37,38]. However, the aforementioned fiber-containing materials only use wood or polymer components.

However, there are many references to the increasing use of natural plant fibers in geopolymer or cement composites [39,40,41,42]. The use of natural plant fibers in geopolymer and cement composites is a growing trend in the field of construction materials. Such fibers are attractive due to their ecological, mechanical and economic properties. The demand for energy-efficient building and industrial materials has stimulated the development of composites containing natural fibers and more environmentally friendly matrices. Natural fibers have many advantages: biodegradability, renewability, low density, high specific strength and low cost [43,44,45]. Geopolymers are also of increasing interest because they are produced at low temperatures and their main building blocks are industrial wastes: fly ash (a by-product from coal-fired power plants) or metakaolin (an inorganic aluminosilicate material). The utilization of natural plant fibers in geopolymer and cement composites is a promising and rapidly growing field that combines material innovation with environmental care [46,47,48].

The trend of using natural fibers in building materials has gained significant momentum in recent years, driven by the increasing demand for sustainable and eco-friendly construction practices. Natural fibers, such as jute, sisal, hemp, and kenaf, are being explored as viable alternatives to synthetic fibers due to their abundant availability, low cost, and favorable mechanical properties. These fibers are not only renewable but also biodegradable, making them an attractive option for environmentally conscious construction projects [49,50].

Research indicates that the incorporation of natural fibers into composite materials can enhance their mechanical properties while reducing the overall weight of the structures [51,52]. For instance, studies have shown that composites reinforced with natural fibers exhibit comparable strength to those reinforced with synthetic fibers, such as glass or carbon fibers, but with a significantly lower environmental impact [53,54]. The use of natural fibers in building materials also contributes to improved thermal and acoustic insulation, which is essential for energy-efficient construction [55].

Moreover, the versatility of natural fibers allows for their application in various forms, including insulation boards, particle boards, and structural components [49,56]. The combination of natural fibers with polymers in hybrid composites has been particularly effective in enhancing the performance of building materials. For example, the blending of different natural fibers with resin has been shown to optimize the mechanical properties of the resulting composites, making them suitable for a wide range of applications in the construction industry [54,57].

The growing interest in natural fibers is also reflected in the increasing number of studies focused on their mechanical performance and potential applications. Researchers are exploring innovative methods to improve the processing and integration of natural fibers into composite materials, addressing challenges such as fiber orientation and compatibility with matrix materials [53,58]. As a result, the trend towards using natural fibers in building materials is expected to continue, driven by advancements in material science and a collective push towards sustainability in the construction sector [59].

In conclusion, the trend of utilizing natural fibers in building materials is characterized by a shift towards sustainable practices, enhanced mechanical performance, and a growing body of research aimed at optimizing their use. This movement not only addresses environmental concerns but also opens new avenues for innovation in the construction industry.

The aim of this publication was to carry out physical tests for eco-friendly layered geopolymer materials. The surface material of the new composite was a geopolymer concrete based on F-grade fly ash and quartz sand, obtained by alkaline activation of aluminosilicates. Plant-based components of natural origin—coconut mat and flax and hemp non-woven fabric—were used as the core material. The building envelope was created through a pouring method, where two types of fiber were placed inside as natural insulation and composite reinforcement. The incorporation of natural fibers such as coconut mats, flax/hemp nonwoven fabrics, and composite rods into geopolymer composites is justified by several compelling factors, including sustainability, mechanical performance, and thermal properties. These natural fibers contribute to the overall performance of geopolymer composites while aligning with the growing emphasis on environmentally friendly construction materials. Coconut mats, derived from the fibrous husk of coconuts, exhibit excellent mechanical properties and low density, making them suitable for use as reinforcement in geopolymer composites. Research has shown that the addition of coconut fibers can significantly enhance the flexural strength of geopolymer matrices, thereby improving their structural integrity. Furthermore, coconut fibers are abundant and biodegradable, which aligns with sustainable development goals by reducing reliance on synthetic materials and minimizing environmental impact. The use of coconut mats also contributes to the reduction in waste, as they are often by-products of coconut processing. Flax and hemp nonwoven fabrics are also gaining traction in geopolymer composites due to their high tensile strength and lightweight characteristics. These fibers have been shown to improve the mechanical properties of geopolymer composites, including tensile strength and impact resistance. The natural origin of flax and hemp fibers not only provides a renewable resource but also enhances the thermal insulation properties of the composites, making them suitable for energy-efficient building applications. Additionally, the alkali resistance of these fibers can be improved through chemical treatments, further enhancing their compatibility with the alkaline environment of geopolymer matrices. Composite rods, often made from a combination of natural fibers and polymers, offer another layer of reinforcement in geopolymer composites. Their incorporation can lead to improved load-bearing capacity and durability under various environmental conditions. The use of composite rods allows for tailored mechanical properties, as the fiber orientation and composition can be adjusted to meet specific structural requirements. This adaptability is particularly beneficial in construction applications where varying load conditions and environmental factors are present. The choice of coconut mats, flax/hemp nonwoven fabrics, and composite rods in geopolymer composites is justified by their mechanical performance, sustainability, and thermal properties. These materials not only enhance the structural integrity of geopolymer composites but also contribute to environmentally friendly construction practices, making them a valuable addition to modern building materials.

This proposed new building material solution will be a much more modern, energy-saving and environmentally friendly option than any previous concept of using traditional structural materials. The advantages of geopolymer building envelopes with natural fibers include:

1. Ecology: Natural fibers are renewable, biodegradable and have a low carbon footprint. Fly ash is a waste raw material with a lower carbon footprint and the emissions of CO_2_ and other greenhouse gases during its production are up to 10 times lower.
2. Energy efficiency: In the production of geopolymers, 4–8 times less electricity is used than in traditional building materials. Natural fibers have lower embedded energy and lower embedded carbon dioxide by up to 5–10 times.
3. Health: no harmful chemicals, which improves indoor air quality.
4. Insulation: excellent thermal and acoustic insulation properties.
5. Durability: Natural fibers are resistant to mold, fungi and moisture, which increases the durability of building materials. Geopolymer concretes have excellent mechanical properties.

The immediate goal of this research was to confirm the feasibility of producing geopolymer composites with insulating layers made of renewable materials, which would be characterized by compressive strengths like those of C25/30-grade concrete and thermal conductivity coefficients like those of lightweight concrete. The goal of achieving high flexural strength was achieved by using reinforcement in the form of composite bars with FGRC. The research hypothesis the authors of this paper set themselves was as follows: It is possible to produce environmentally friendly composites for use in construction as both structural and insulating materials, manufactured using renewable materials and characterized by strengths corresponding to conventional structural concretes (min. 25 MPa) and with a reduced thermal conductivity coefficient corresponding to parameters for lightweight concretes (max. 0.8 W/m·K).

The presented research results are a new contribution to the development of environmentally friendly building materials, contributing to the development of sustainable construction. The possibilities of using natural fibers in construction have been studied for years; however, the described solutions of layered composites with multiple fiber insulation layers have essentially not been researched. This highlights the novelty of this research and the results obtained are very attractive and may inspire other scientists to develop this concept and conduct further research in this area.

## 2. Materials and Methods

**Figure 1:**
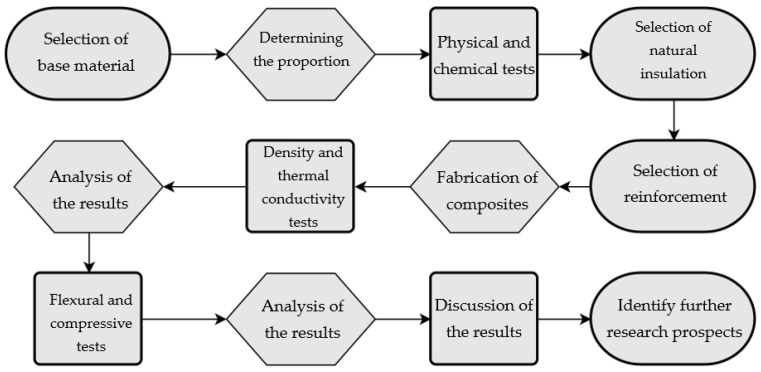
Flowchart of the steps followed in this study.

For the purpose of understanding the methodology process in this paper, an experimental flowchart of the steps followed in the method and research parts is provided below in Figure 1.

### 2.1. Base Materials

The layered geopolymer composites were produced on the basis of fly ash from the Skawina CHP Plant (Skawina, Poland) and quartz sand from the Skawina Sand Plant (Świętochłowice, Poland). The F-grade fly ash used in this study is characterized by a high content of silicon dioxide and more than half as much diglinium trioxide. The quartz sand, on the other hand, is characterized by a very high SiO_2_ content of approximately 90 wt.%. The geopolymer concrete was made by adding both components in a 50/50 weight ratio. Polycondensation of the geopolymer was initiated by adding a 10 mol alkaline solution of sodium silicate with sodium water glass. Sodium silicate R-145 with a molar modulus of 2.5 and a density of approximately 1.45 g/cm^3^ was used to produce the geopolymer concrete.

### 2.2. Natural Insulating Materials

Natural insulating materials, typically used in the furniture and upholstery industry, were used to produce layered geopolymer composites. Several layers of two different natural insulating materials were introduced into the composite as the core material. Coconut mats sourced from the upholstery wholesaler Akces (Strażów, Poland) and flax and hemp non-woven fabrics produced by Double Raw Fiberworks (Kraków, Poland) were used in the study. The coconut mat was made from natural fibers obtained from the coconut shell, which had undergone suitable processing to ensure their durability over a long period of time. The thickness of one mat was 1 cm and the weight was 1200 g/m^2^. The flax and hemp non-woven fabric was produced by mechanical needling. The proportion of flax and hemp fibers was 50/50 wt.%. The thickness of a single nonwoven was approximately 5 mm and the grammage was 750 g/m^2^.

### 2.3. Reinforcement

The finished structural element was reinforced with glass fiber and epoxy resin composite bars (Trokotex Polymer Group Ltd., Toruń, Poland). Bars with diameters of 4 and 8 mm were used to compare the effect of the reinforcement on the physical properties of the composite. Composite bars for concrete reinforcement are a new, innovative and more economical technology, due to the fact that they do not require costly maintenance and repairs like reinforced concrete structures after a long service life. Glass fiber reinforcement is completely corrosion-resistant and, importantly, due to its use in the production of geopolymer concrete, it does not change its properties even in highly corrosive and aggressive acid or alkaline environments. The bars are also characterized by high frost resistance and up to three times longer material life. The bars are nine times lighter (with the same strength) and therefore have lower transport costs and a lower unit price. The composite reinforcement’s conductivity rating is a hundred times lower than that of steel; so, it does not cause heat bridging and saves on the building’s running expenses.

### 2.4. Eco-Friendly Layered Geopolymer Composites

**Figure 2:**
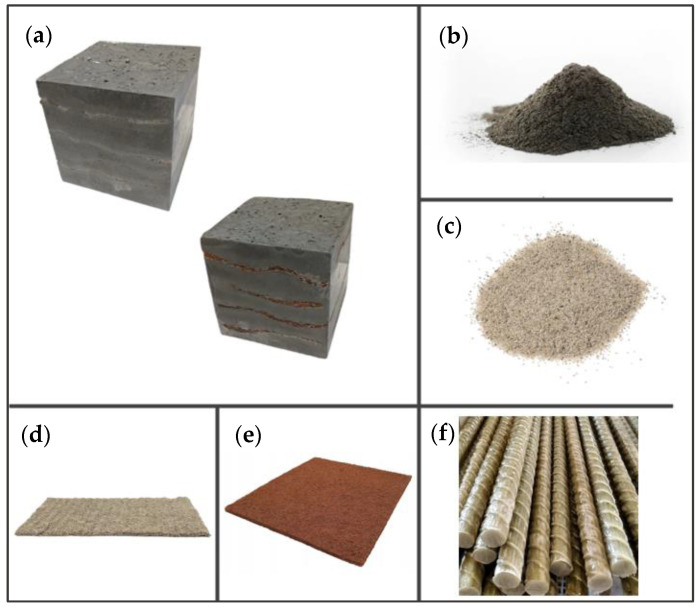
Eco-friendly layered geopolymer composites: (**a**) ready composites, (**b**) fly ash, (**c**) sand, (**d**) flax-hemp trawl, (**e**) coconut mat, (**f**) composite bars.

Due to the use of lower-carbon-footprint materials and alternative components to traditional construction in the layered geopolymer composite, it was decided to designate the composite environmentally friendly and sustainable. Anthropogenic waste from the combustion of coal in power and thermal power plants was used as the main base material. The commonly used polycyclic isocyanates were replaced exclusively with natural biodegradable insulation. An alternative to steel and reinforced concrete—innovative composite bars—was also used to reinforce the composite. The two insulating materials were selected due to their similar density and thermal conductivity, along with their wide availability in the market. While coconut mat is commonly used in mattresses, its application as insulation in building envelopes has not been explored, prompting the idea of using it in a different industry. The second material shares similar characteristics with coconut mat, leading to a comparison of their performance in multilayer partitions. As previously mentioned, the multilayer geopolymer composites were created using fly ash and sand, with rebar added to strengthen the prefabricated prototype. Composite reinforcement was chosen for several key reasons, as it offers an effective structural solution when used with multilayer natural fiber mats and felts. Its thermal expansion is similar to that of concrete, preventing cracks in concrete due to temperature fluctuations, thus avoiding repair costs during the building’s lifespan. Fiberglass reinforcement is completely resistant to corrosion, maintaining its properties even in harsh acidic and alkaline environments, and does not require costly maintenance. Additionally, these rods exhibit high frost resistance and offer a material lifespan 2–3 times longer than that of conventional options. Nine reinforcing bars with a length of 15 cm and diameters of 4 and 8 mm were added to each layered geopolymer composite. Two thicknesses of reinforcement were used to compare the effects on the strength and insulation of the finished composites. The composites also included 4 and 8 core layers and the maximum amount of component, i.e., 10 and 12 layers of natural insulation. Due to the different thicknesses and weights of the insulation, a different weight amount of components was introduced into the layered composite. The composites were manufactured using the pouring method. In the mold, a layer of base material and a core were laid alternately. Reinforcing bars were placed at equal distances in an amount of 9 pieces. The reinforcement in the composite was arranged symmetrically at regular spacing. The specimens were then cured in an SLW 750 laboratory dryer at 75 °C and unmolded after 24 h. Each composite had dimensions of 150 × 150 × 150 mm. All samples were produced under laboratory conditions at 23 °C in 40–60% humidity. Samples for density and conductivity tests were dried to dry weight, while samples for strength tests were conditioned for 28 days under laboratory conditions. For density and thermal conductivity tests, each cube was cut into 3 150 × 150 × 50 mm slabs. Three samples of each type were used for each test. For flexural strength tests, each slab was cut into 3 cuboids measuring 150 × 50 × 50 mm. The test used 3 samples of each type. For the compressive strength tests, the same cuboids were used using an additional two 50 × 50 mm metal plates. Six samples of each type were used for the test. All results of this study were statistically analyzed. For each test, the standard deviation was added, and for the strength results, error bars were plotted on graphs. In addition to the various composites, a reference sample labeled “R.G.” was produced. Figure 2 shows the layered geopolymer composite and all the components used to produce the material. Table 1 shows the designations of the specimens whose test results will be analyzed later in this manuscript, as well as the weight contribution of the components combined (insulation and reinforcement).

### 2.5. Methods

#### 2.5.1. Density Measurements

Here, the density of the prepared composition sheets was determined by a geometrical method, according to the weight and volumes of the specimens. The regular shaped material densities are defined by the following formula:

$$d=\frac{m}{V}\;[\frac{kg}{m^{3}}]$$

where:

*m*—mass of material [kg];
*V*—volume of material [m^3^].

#### 2.5.2. Flexural Strength Measurements

Testing of the flexural strength was conducted with the MATEST 3000 kN strength machine (Matest, Treviolo, Italy). To determine the flexural strength of cement mortar specimens, the procedure is set out in EN 196-1:2016-07 (Cement test methods—Part 1: Strength reduction—Section 9.1) [60]. Rectangular test specimens are subjected to a bending load by applying an equal loading force caused by the top and bottom rollers in the test system. Testing is carried on further until the maximum loads that cause damage to the component are achieved, and the flexural strength is evaluated from this value. The flexural strength can be calculated with the formula for the three-point test method:

$$R_{f}=\frac{1.5\times F_{f}\times l}{b^{3}}\left[MPa\right]$$

where:

*R_f_*—flexural strength [MPa];
*b*—lateral length of the section [mm];
*F_f_*—maximum load [N];
*l*—length between supports [mm].

#### 2.5.3. Compressive Strength Measurements

Tests for compression resistance were conducted using a MATEST 3000 kN machine (Matest, Treviolo, Italy). The governing document in the construction market is EN 196-1:2016-07 (Methods for testing cement—Part 1: Determination of strength—Section 9.2) [60], which defines the compression test method for cement mortar specimens. This compression test applies a load to the specimens up to a failure point that will cause the material to fracture. These maximum stresses are the basis for calculating the compressive strength for the concrete material following the formula below:

$$R_{c}=\frac{F_{c}}{2500}\left[MPa\right]$$

where:

*R_c_*—compressive strength [MPa];
2500—surface of tiles (or auxiliary tiles) [mm^2^];
*F_c_*—maximum load [N].

#### 2.5.4. Thermal Conductivity Measurements

The thermal conductivity coefficient *λ* (lambda) is the amount of heat energy flowing through a certain mass of a sample as a result of an external temperature difference. In the case of a heat-conductive square material subject to constant conditions, however, the quantity carried is material-dependent, being proportional to the body’s surface cross-sectional areas, the difference in temperature and the time taken for the heat to flow. The amount is given by the formula:

$$\lambda =\frac{Q}{t}\times \frac{d}{S∆T}[\frac{W}{m\cdot K}]$$

where:

*Q*—amount of heat flowing through the body [J];
*t*—flow time [s];
*d*—partition thickness [m];
*S*—cross-sectional area of the body [m^2^];
∆T—temperature difference in the direction of heat conduction [K].

## 3. Results

### 3.1. Tests of Base Materials

Chemical composition analysis of the oxides was conducted for fly ash and sand, the base materials. XRF analysis of the oxides was undertaken with a SCHIMADZU EDX-7200 (SHIMADZU Europa GmbH, Duisburg, Germany). The experiments took place under an air ambient atmosphere using grips developed for bulk materials with Mylar film, and the results are summarized in Table 2. Those oxides with a percentage of more than 0.1 wt.% are displayed in the table below.

Analysis by mineral phase for the basic materials was conducted with a PANalytical AERIS batch machine (Malvern PANalytical, Lelyweg 1, Almelo, The Netherlands). Assays were performed with XRD X-ray diffraction analyzer technology and the results are presented in Table 3. The quantitative approach to the analysis followed the Rietveld method, implemented in HighScore Plus software (version 4.8). During the course of the analysis, the PDF-4+ database provided by the International Centre for Diffraction Data (ICDD) was employed. Measures were registered between 10 and 100° at a step of 0.003° (2θ) with a time per step for 340 s, with the Cu Kα radiation.

**Figure 3:**
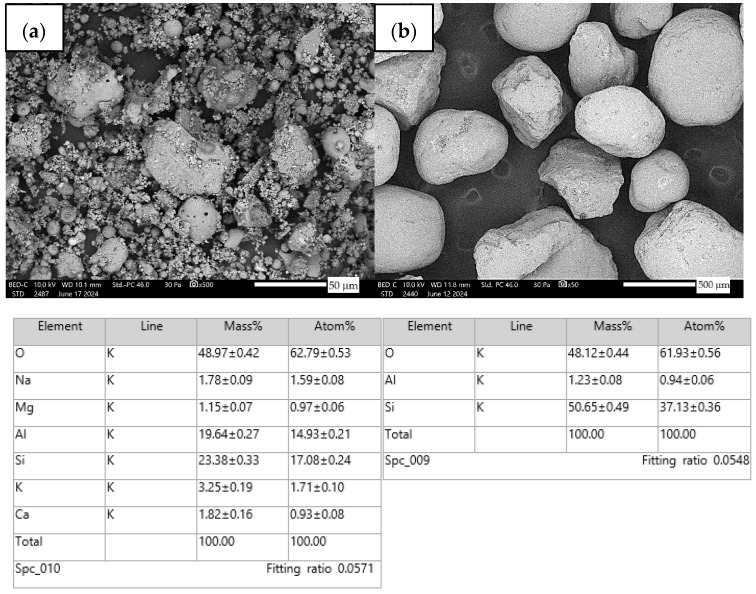
Fly ash morphology and EDS (**a**), sand morphology and EDS (**b**).

SEM images were also obtained for the fly ash and the sand using a JEOL IT 2000 scanning electron microscope (JEOL, Akishima, Tokyo, Japan). In order to carry out the observation with the SEM microscope, the samples were fitted to dedicated charcoal disks and positioned on metallic benches and then placed in a holder. A special EM-Tec C33 carbon adhesive was also used to better fix the material and lead to better material conduction. The surface of the material was treated to a conductive gold layer prior to examination with a vacuum sputtering device, the DII-29030SCTR Smart Coater (JEOL Ltd., Peabody, MA, USA). The authors analyzed the morphology of the samples and studied the elemental composition using EDS. SEM images of the ash were taken at 500× magnification, and of the sand at 50× magnification. Figure 3 shows the results.

Raw materials with high silicon and alumina content for the production of geopolymers were employed. The above chemical composition analysis shows that both fly ash and sand are characterized by a high content of these oxides. Fly ash typically contains 50–60 wt.% SiO_2_ and about half as much Al_2_O_3_. Sand is usually characterized by a high SiO_2_ content (around 90–99 wt.%). Analysis of the mineral phases showed that the fly ash consists of mullite, quartz, hematite, magnetite, anhydrite and rutile. All identified phases are typical of F-grade fly ash. Analysis of the mineral phases of the sand revealed the presence of quartz and calcite—these are the characteristic mineral phases found in quartz sands. Figure 2 shows the morphology of the base materials and the elemental chemical composition. The morphology of the sand and ash is characteristic of this type of material. For the fly ash, magnesium was additionally identified in the composition, but in very small amounts. The oxide, mineralogical and elemental chemical composition of both materials is very similar regardless of the chosen test method. The content of the most important oxides made by the different methods converges. Geopolymers consist of long chains (copolymers) of alumino-silicon and aluminum, stabilized by metal cations, most commonly sodium, potassium, lithium or calcium. Both these materials—fly ash and sand—therefore fit into the concept of geopolymer materials. Both fly ash and sand, when added with an alkaline activator, react very well with each other and show the ability to form a stable and durable structure, similar to Portland cement-based concretes and mortars.

### 3.2. Density of Eco-Friendly Layered Geopolymer Composites

The density of cut slabs of layered geopolymer composites was determined as an average of three measurements. Each slab was characterized by a similar arrangement of layers; so, it was decided to average this value. The results are presented in Table 4. The volume of the slabs was constant (150 × 150 × 50 mm), but the weight tended to vary by a few grams from sample to sample. As the number of added components increased, the differences in weight measurements varied by up to a few tens of grams, due to the technological difficulty of placing the layers evenly. Table 3 additionally includes the standard deviation for the density measurement. Specimen measurements were made using a laboratory caliper to an accuracy of 0.01 mm and the mass of the test samples was quantified with a RADWAG PS 200/2000.R2 (RADWAG Electronic Scales, Radom, Poland) laboratory precision weighing instrument to an error of 0.01 g.

Each component added to the layered geopolymer composite resulted in a decrease in density due to the fact that the introduced additives reduced the proportion of geopolymer concrete in relation to the natural insulation and reinforcement. The largest decrease in density was observed for slabs 10C_R8 and 12F-H_R8 (41% decrease). These were slabs in which the maximum amount of natural insulation was placed. As for the composites with eight layers, a reduction in density of 21% was obtained for the 8F-H_R4 composite and 35% for the 8C_R4. As for the composites with four layers of insulation, a greater decrease in density was always observed in composites with larger diameter reinforcement because the larger surface area of the bars resulted in a reduction in the proportion of geopolymer concrete. For four layers of natural insulation, the density decreased by up to 19%—4C_R8. The coconut mats were of a heavier weight than the flax/hemp non-woven fabrics and therefore caused a greater decrease in density in each composite. The density results obtained are comparable to the properties of lightweight concretes (800 kg/m^3^ to 2000 kg/m^3^).

### 3.3. Flexural Strength of Eco-Friendly Layered Geopolymer Composites

**Figure 4:**
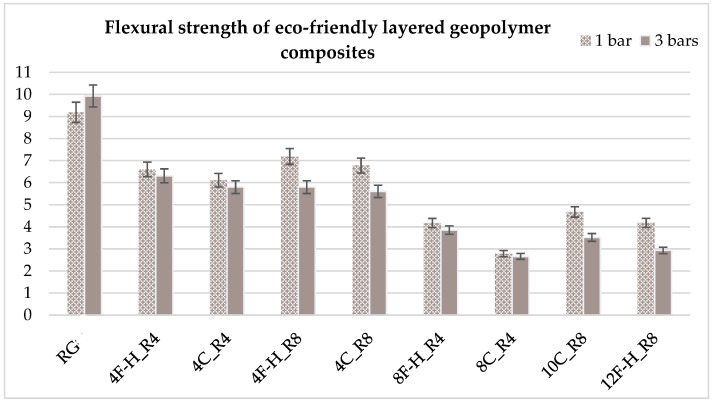
Comparison of flexural strength results of eco-friendly layered geopolymer composites.

For the flexural strength tests, the slabs that were tested for density and thermal conductivity were cut into three rectangular specimens measuring 150 × 50 × 50 mm. For the tests, two slabs were used and cut in two different directions—the first option being a cut along the bars, while the second was a cut across the bars. In view of this, six specimens were created to determine the flexural strength—three specimens with one long bar along the perpendicular, and three specimens with three short bars of 50 mm in length. The flexural strength R_f_ was determined as the average value of the three measurements. Table 5 and Table 6 show the results of the tests, together with photographs of the test specimens. Figure 4, on the other hand, compares the effect of bar arrangement in the composite in terms of bending strength.

The highest flexural strength was found in the specimen without the addition of reinforcement and natural insulation—around 10 MPa. In each case, the addition of layers of insulation resulted in a decrease in flexural strength, but with the addition of reinforcement, the strength results are quite high. Each specimen that had one bar in the center of the cuboid had a higher strength rating compared to the cuboids with three short bars, but the differences are not very high because in each of the composite variants, the bar was placed in the center and this is where the material breaks. Of the fiber-reinforced composite variants, specimens 4F-H_R8 and 4C_R8 had the highest flexural strengths—around 7 MPa (one bar) and around 6 MPa (three short bars). The worst results were obtained for composites 8F-H_R4—about 4 MPa—and 8C_R4—about 3 MPa. Composites in which there was a maximum amount of natural insulation obtained similar strength results in relation to the eight core layers (from 3–4.5 MPa). However, each result shows that the strength of all composites is within the recommended strength parameters for concrete and building envelope. The flexural strength results obtained are comparable to the properties of C25/30 concretes.

### 3.4. Compressive Strength of Eco-Friendly Layered Geopolymer Composites

**Figure 5:**
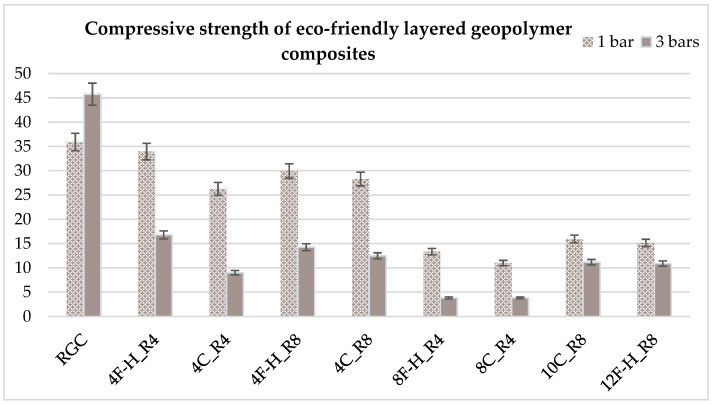
Comparison of compressive strength results of eco-friendly layered geopolymer composites.

For the compressive strength tests, rectangular bending test specimens were used. Two metal plates measuring 50 × 50 mm were applied to the ends of the cuboids from above and below, and the strength parameters of the composites were tested. All the specimens that had been tested for flexural strength were used for testing. Again, two variations in bar arrangement were used—six specimens with one long bar along the perpendicular, and six specimens with three short bars of 50 mm in length. The compressive strength R_c_ was determined as an average value from four measurements (the two outermost measurements were discarded due to the large scatter of R_c_ values). Table 7 and Table 8 show the results of the tests, together with photographs of the test specimens. Figure 5, on the other hand, compares the effect of bar arrangement in the composite in terms of compressive strength.

The highest compressive strength was again found in the sample without the addition of reinforcement and natural insulation, with an average of 40 MPa. In each case, the addition of layers of insulation resulted in a decrease in compressive strength, but with the addition of reinforcement, the strength results for most specimens exceeded 15 MPa. Each specimen that had one bar in the center of the cuboid had a higher strength index compared to the cuboids with three short bars, and for this study, the differences are already very large. Of the fiber-reinforced composite variants, specimens 4F-H_R4 and 4F-H_R8 had the highest compressive strengths of about 30 MPa (one bar) and about 15 MPa (three short bars). The worst results were obtained for composites 8F-H_R4 and 8C_R4—about 10 MPa for samples with one bar and about 5 MPa for samples with three short bars. Composites in which there was a maximum amount of natural insulation obtained similar strength results—around 15 MPa for samples with one bar and around 10 MPa for samples with three short bars. However, each result shows that the strength of all composites is at a good level and is within the range of recommended strength parameters for concrete and building envelope. The results obtained for compressive strength are comparable to the properties of C25/30 concretes.

### 3.5. Thermal Conductivity of Eco-Friendly Layered Geopolymer Composites

Conductivity coefficient tests were conducted with a Lambda HFM 446 plate instrument (Netzsch, Selb, Germany). The apparatus functions to standards such as ISO 8301, EN 12664, ASTM C1784, ASTM C518 [61,62,63,64] and similar ones. Temperature regulation and control is verified by a Peltier system. Thermally relevant features for the fabricated panels were identified with the described apparatus, which was based upon the hot and cold plate approach. The thermal conductivity was tested at 0–20 °C. Table 9 shows the average conductivity based on the three tests.

Each component added to the layered geopolymer composite resulted in a decrease in the thermal conductivity coefficient due to the fact that the introduced additives reduced the proportion of geopolymer concrete in relation to the natural insulation and reinforcement, which has a very low thermal conductivity coefficient. The largest decrease in the thermal conductivity coefficient was observed for the 10C_R8 and 12F-H_R8 slabs (a decrease of approximately 60%). These were slabs in which the maximum amount of natural insulation was placed. For the composites with eight layers, a reduction in lambda coefficient of 19% was obtained for the 8F-H_R4 composite and 42% for the 8C_R4. As for composites with four layers of insulation, the largest decrease in thermal conductivity coefficient was observed for the 4C_R4 panel—a decrease of 23%. For composites with four layers of insulation and 8 mm reinforcement, a decrease in lambda of approximately 10% was observed. In each specimen with larger diameter reinforcement, a decrease in the tested parameter was observed due to the reduction in the proportion of geopolymer concrete in the composite in favor of natural insulation. The coconut mats were heavier than the flax and hemp non-woven fabrics and therefore caused a greater decrease in lambda in each composite. The thermal conductivity results obtained are similar to those of lightweight concretes (from 0.35 W/(m·K) for density class D1.0 to 0.8 W/(m·K) for class D1.8).

## 4. Discussion

The present study analyzed the physicochemical properties of the base materials and the physical, mechanical and insulating properties of the organic layered geopolymer composites. The primary aim of this research was to validate the potential for creating geopolymer composites with insulating layers made from renewable materials. These composites were intended to have compressive strengths comparable to C25/30-grade concrete and thermal conductivity similar to lightweight concrete. High flexural strength was achieved by incorporating composite bars reinforced with FGRC. The authors hypothesized that it is possible to develop environmentally friendly composites for construction, serving both structural and insulating purposes, made from renewable materials and offering strength equivalent to that of standard structural concretes (at least 25 MPa) while also having lower thermal conductivity, in line with lightweight concrete standards (maximum 0.8 W/m·K).

Investigations of the physicochemical properties of fly ash and sand showed that the base materials are characterized by a high content of silicon and aluminum oxides. Fly ash typically contains 50–60 wt.% SiO_2_ and about half as much Al_2_O_3_. Sand is usually characterized by a high SiO_2_ content (around 90–99 wt.%). Analysis of the mineral phases showed that fly ash consists of mullite, quartz, hematite, magnetite, anhydrite and rutile. All identified phases are typical of F-grade fly ash. Analysis of the mineral phases of the sand revealed the presence of quartz and calcite—these are the characteristic mineral phases found in quartz sands. The morphology of the sand and ash is characteristic of this type of material. The oxide, mineralogical and elemental chemical composition of both materials is very similar regardless of the chosen test method. The content of the most important oxides made by the different methods converges. The results of other researchers confirm the above research conclusions [65,66,67].

Investigations into the physical properties of the organic layered geopolymer composites showed that each added component caused a decrease in density due to the fact that the introduced additives reduced the proportion of geopolymer concrete in relation to the natural insulation and reinforcement. The largest decrease in density was observed for slabs 10C_R8 and 12F-H_R8 (41% decrease). These were slabs in which the maximum amount of natural insulation was placed. For the composites with eight layers, a reduction in density of 21% was seen for the 8F-H_R4 composite and 35% for the 8C_R4. With regard to composites with four layers of insulation, a greater decrease in density was always observed in composites with larger diameter reinforcement because the larger surface area of the bars resulted in a reduction in the proportion of geopolymer concrete. For four layers of natural insulation, the density decreased by up to 19%—4C_R8. The coconut mats had a higher grammage than the flax/hemp non-woven fabrics and therefore caused a greater decrease in density in each composite. The decrease in density in cementitious and geopolymer composites following the introduction of lower density components has been confirmed more than once in the literature. This effect is a natural physical phenomenon often observed by researchers. The resulting densities are comparable to those of lightweight concretes (800 kg/m^3^ to 2000 kg/m^3^) [68,69,70,71,72].

Analysis of the mechanical properties showed that the highest flexural strength was that of the specimen without the addition of reinforcement and natural insulation—around 10 MPa. In each case, the addition of layers of insulation resulted in a decrease in flexural strength, but with the addition of reinforcement, the strength results are quite high. Each specimen that had one bar in the center of the cuboid had a higher strength rating compared to the cuboids with three short bars, but the differences are not very high because in each of the composite variants, the bar was placed in the center and this is where the material breaks. Of the fiber-reinforced composite variants, specimens 4F-H_R8 and 4C_R8 had the highest flexural strengths—around 7 MPa (one bar) and around 6 MPa (three short bars). The worst results were obtained for composites 8F-H_R4—about 4 MPa and 8C_R4—about 3 MPa. Composites in which there was a maximum amount of natural insulation obtained similar strength results in relation to the 8 core layers (3–4.5 MPa). Other researchers, also observed that the addition of natural fibers to the composites resulted in a decrease in flexural strength. The results of other researchers overlap with the results presented in this paper and were typically around 5 MPa. Flexural strength results are comparable to those of C25/30 concretes [46,73,74,75].

Analysis of the mechanical properties in terms of compressive strength revealed that the highest compressive strength was again that of the sample without the addition of reinforcement and natural insulation, with an average of 40 MPa. In each case, the addition of layers of insulation resulted in a decrease in compressive strength, but with the addition of reinforcement, the strength results for most specimens exceed 15 MPa. Each specimen that had one bar in the center of the cuboid had a higher strength index compared to the cuboids with three short bars, and for this study the differences are already very large. Of the fiber-reinforced composite variants, specimens 4F-H_R4 and 4F-H_R8 had the highest compressive strengths of about 30 MPa (one bar) and about 15 MPa (three short bars). The worst results were obtained for composites 8F-H_R4 and 8C_R4—about 10 MPa for samples with one bar and about 5 MPa for samples with three short bars. Composites in which there was a maximum amount of natural insulation obtained similar strength results—about 15 MPa for specimens with one bar and about 10 MPa for specimens with three short bars. Again, the decrease in compressive strength parameters after the addition of low-density components has already been observed more than once. The results for compressive strength are quite similar as far as the work of other researchers is concerned and on average settle at 20 MPa. The performance results for compressive strength are comparable to those of C25/30 concretes [74,76,77].

The last parameter already analyzed was the thermal conductivity coefficient. Each component added to the layered geopolymer composite resulted in a decrease in the thermal conductivity coefficient due to the fact that the introduced additives reduced the proportion of geopolymer concrete in relation to the natural insulation and reinforcement, which has a very low thermal conductivity coefficient. The largest decrease in thermal conductivity coefficient was observed for the 10C_R8 and 12F-H_R8 slabs (a decrease of approximately 60%). These were slabs in which the maximum amount of natural insulation was placed. For the composites with eight layers, a reduction in lambda coefficient of 19% was obtained for the 8F-H_R4 composite and 42% for the 8C_R4. As for composites with four layers of insulation, the largest decrease in thermal conductivity coefficient was observed for the 4C_R4 panel—a decrease of 23%. For composites with four layers of insulation and 8 mm reinforcement, a decrease in lambda of approximately 10% was observed. In each specimen with larger diameter reinforcement, a decrease in the tested parameter was observed due to the reduction in the proportion of geopolymer concrete in the composite in favor of natural insulation. The coconut mats had a higher grammage than the flax and hemp non-woven fabrics and therefore caused a greater decrease in lambda in each composite. The decrease in thermal conductivity following the introduction of lower density components has been observed by other researchers on more than one occasion. The decreases in density and thermal conductivity are closely related and are natural physical phenomena. The thermal conductivity results are similar to those of lightweight concretes (from 0.35 W/(m·K) for density class D1.0 to 0.8 W/(m·K) for class D1.8) [78,79,80].

The research results presented offer a fresh contribution to the advancement of eco-friendly building materials, supporting the growth of sustainable construction. While the use of natural fibers in construction has been explored for many years, the innovative approach of layered composites with multiple fiber insulation layers is virtually unheard of. This underscores the novelty of this study, making the findings particularly compelling and likely to inspire other researchers to expand on this concept and pursue further investigation in this field.

The competitive advantage of these geopolymer composites over traditional materials lies in their performance properties and versatility. In addition, the low synthesis temperatures of geopolymers make them compatible with a variety of organic fibers, allowing for a wider range of reinforcement options compared to conventional composites, which typically rely on synthetic fibers such as glass or carbon. This adaptability not only improves properties but also allows for the development of lightweight and high-performance materials that can meet the demands of modern engineering applications. Geopolymer composites are a promising alternative to traditional materials. Their superior properties, durability and versatility make them competitive in the materials market, paving the way for innovative applications in construction and other industries.

## 5. Conclusions

Based on the research results presented in this article, several conclusions can be drawn to summarize this research work:

I. The largest decrease in density was observed for slabs 10C_R8 and 12F-H_R8 (41% decrease). These were the slabs in which the maximum amount of natural insulation was placed. For composites with eight layers, a reduction in density of 21% was obtained for the 8F-H_R4 composite and 35% for 8C_R4. Densities are comparable to those of lightweight concretes (800 kg/m^3^ to 2000 kg/m^3^).
II. Among the variants of composites with fibers and reinforcement, the highest flexural strengths were for specimens 4F-H_R8 and 4C_R8—about 7 MPa (one bar) and about 6 MPa (three short bars). The worst results were obtained for composites 8F-H_R4—about 4 MPa—and 8C_R4—about 3 MPa. The flexural strength results are comparable to those of C25/30 concretes.
III. The highest compressive strengths were for samples 4F-H_R4 and 4F-H_R8—about 30 MPa (one bar) and about 15 MPa (three short bars). The worst results were obtained for composites 8F-H_R4 and 8C_R4—about 10 MPa for samples with one bar and about 5 MPa for samples with three short bars. The compressive strength results are comparable to those of C25/30 concretes.
IV. The largest decrease in thermal conductivity coefficient was observed for 10C_R8 and 12F-H_R8 panels (a decrease of about 60%). These were slabs in which the maximum amount of natural insulation was placed. In the case of composites with eight layers, a decrease in the lambda coefficient of 19% was observed for the 8F-H_R4 composite and a decrease of 42% was observed for the 8C_R4 composite. The thermal conductivity results are similar to those of lightweight concretes (from 0.35 W/(m·K) for density class D1.0 to 0.8 W/(m·K) for class D1.8).

From the above test findings, it is clear that the measurements of the density and conductivity are intimately related, a fact that has been repeatedly acknowledged by other researchers and in specialist publications. The best effect on these two parameters was the highest possible amount of natural insulation (10C_R8 and 12F-H_R8 boards). As for the analysis of strength parameters, the effect is quite the opposite. The greatest strength was characterized by samples in which the natural insulation was as little as possible and reinforcement with a larger diameter was introduced. The optimal solution in terms of strength and isolation are therefore samples with the maximum amount of natural isolation, but the tests in terms of strength should be supplemented by measurements of cubes with dimensions of 150 × 150 × 150 mm. Layered geopolymer composites based on fly ash and quartz sand with the addition of layers of natural insulation of plant origin are a novelty in the world of materials engineering; so, the topic will be continued in the authors’ future works. The idea presented in this article has a very high potential for commercialization.

## Figures and Tables

**Table 1:** Determination of eco-friendly layered geopolymer composites.

Type of Composite	ID	Component Share [wt.%]
reference without reinforcement	RG	0
4 flax/hemp non-wovens_R: 4 mm	4F-H_R4	2
4 coconut mats_R: 4 mm	4C_R4	4
4 flax/hemp non-wovens_R: 8 mm	4F-H_R8	3
4 coconut mats_R: 8 mm	4C_R8	6
8 flax/hemp non-wovens_R: 4 mm	8F-H_R4	4
8 coconut mats_R: 4 mm	8C_R4	7
10 coconut mats_R: 8 mm	10C_R8	12
12 flax/hemp non-wovens_R: 8 mm	12F-H_R8	9

**Table 2:** Oxide analysis for base material.

Precursor		Oxide Composition (wt.%)					
	SiO_2_	Al_2_O_3_	Fe_2_O_3_	CaO	K_2_O	TiO_2_	SO_3_
Fly ash	59.21	31.05	3.88	2.29	2.07	0.77	0.50
Sand	98.57	-	0.30	0.36	0.42	-	0.24

**Table 3:** Mineral phase analysis for base material.

Identified Phase	Chemical Formula	Percentage Share [wt.%]
Fly Ash		
Mullite	Al_6_Si_2_O_13_	51.4
Quartz	SiO_2_	43.9
Hematite	Fe_2_O_3_	1.5
Magnetite	Fe_3_O_4_	0.1
Anhydrite	CaSO_4_	2.1
Rutile	TiO_2_	1.1
Sand		
Quartz	SiO_2_	99.7
Calcite	CaCO_3_	0.3

**Table 4:** Density of eco-friendly layered geopolymer composites.

ID	3 Measurements	Mean Density	Standard Deviation	Decrease in Density
	[kg/m^3^]	[kg/m^3^]	[kg/m^3^]	[%]
RG	1710.09; 1733.00; 1706.09	1716.39	11.86	-
4F-H_R4	1641.09; 1637.86; 1654.82	1644.59	7.35	↓4
4C_R4	1448.23; 1406.61; 1381.50	1412.11	27.52	↓18
4F-H_R8	1550.30; 1524.21; 1523.58	1532.70	12.45	↓11
4C_R8	1393.32; 1396.73; 1402.53	1397.53	3.80	↓19
8F-H_R4	1413.16; 1316.76; 1319.69	1349.87	44.77	↓21
8C_R4	1163.89; 1070.37; 1111.89	1115.38	38.26	↓35
10C_R8	1074.78; 943.66; 1034.59	1017.68	54.85	↓41
12F-H_R8	982.70; 980.41; 1066.81	1009.97	40.20	↓41

**Table 5:** Flexural strength of eco-friendly layered geopolymer composites (one bar).

One Bar through the Middle			
ID	R_f_ [MPa]	Avg. R_f_ [MPa]	Photo
RG referencyjna	7.686; 11.015; 8.861	9.187	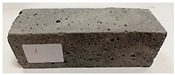 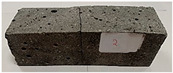 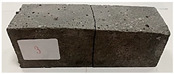
4F-H_R4	6.152; 6.761; 6.893	6.602	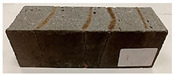 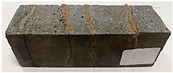 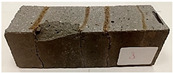
4C_R4	5.565; 6.022; 6.749	6.112	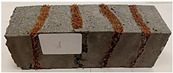 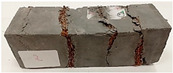 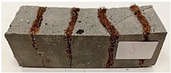
4F-H_R8	5.421; 7.442; 8.698	7.187	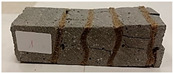 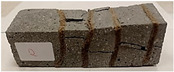 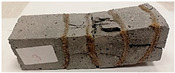
4C_R8	7.458; 6.723; 6.152	6.778	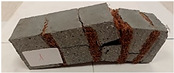 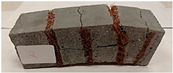 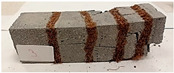
8F-H_R4	3.905; 3.802; 4.807	4.171	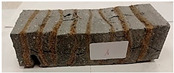 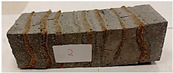 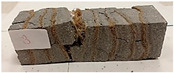
8C_R4	2.627; 2.401; 3.326	2.785	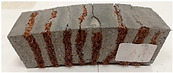 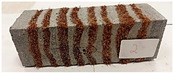 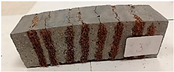
10C_R8	4.569; 4.047; 5.402	4.673	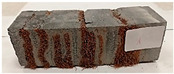 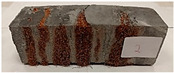 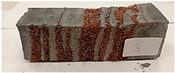
12F-H_R8	4.032; 4.129; 4.374	4.178	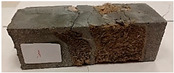 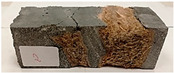 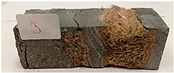

**Table 6:** Flexural strength of eco-friendly layered geopolymer composites (three bars).

Three Short Bars			
ID	R_f_ [MPa]	Avg. R_f_ [MPa]	Photo
RG referencyjna	9.139; 9.726; 10.917	9.927	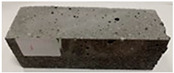 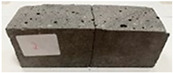 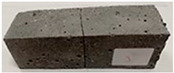
4F-H_R4	5.043; 6.462; 7.425	6.310	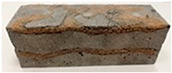 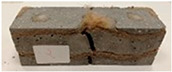 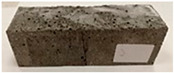
4C_R4	4.618; 5.141; 7.637	5.799	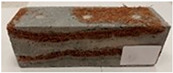 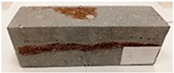 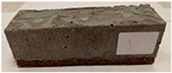
4F-H_R8	4.374; 6.609; 6.413	5.799	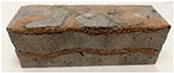 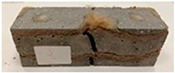 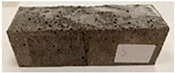
4C_R8	5.287; 5.369; 6.169	5.608	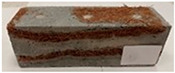 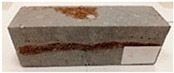 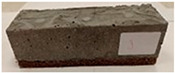
8F-H_R4	3.737; 4.553; 3.264	3.851	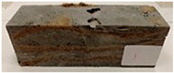 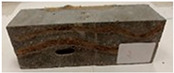 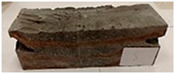
8C_R4	2.281; 2.383; 3.317	2.660	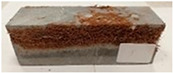 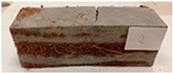 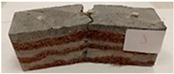
10C_R8	3.394; 2.742; 4.422	3.519	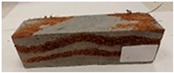 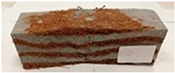 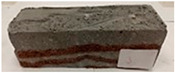
12F-H_R8	4.308; 2.383; 2.109	2.933	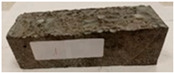 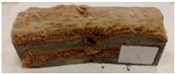 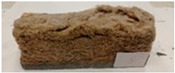

**Table 7:** Compressive strength of eco-friendly layered geopolymer composites (one bar).

One Bar through the Middle			
ID	R_c_ [MPa]	Avg. R_c_ [MPa]	Photo
RG referencyjna	34.477; 31.470; 40.764; 23.357; 38.270; 39.467	35.921	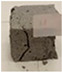 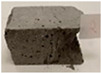 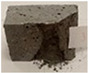 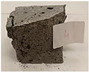 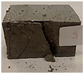 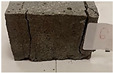
4F-H_R4	35.616; 38.609; 29.895; 37.342; 31.334; 31.462	33.939	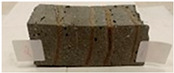 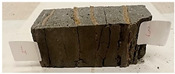 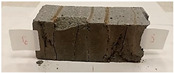
4C_R4	28.056; 18.945; 22.704; 30.840; 28.745; 25.550	26.264	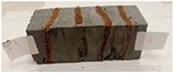 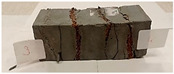 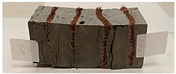
4F-H_R8	29.598; 16.844; 31.378; 22.241; 36.528; 37.638	29.936	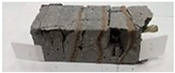 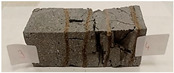 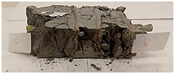
4C_R8	34.448; 10.180; 32.294; 24.844; 21.585; 35.781	28.293	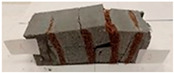 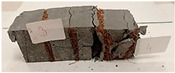 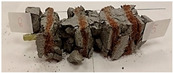
8F-H_R4	18.675; 10.633; 14.905; 6.966; 10.560; 17.260	13.340	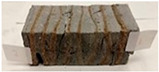 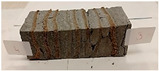 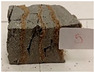 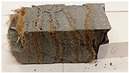
8C_R4	9.048; 21.264; 13.406; 8.062; 9.070; 12.480	11.001	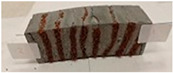 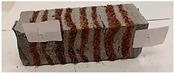 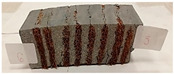
10C_R8	27.426; 4.519; 21.450; 22.916; 10.966; 8.395	15.932	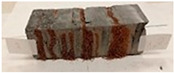 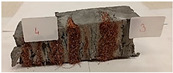 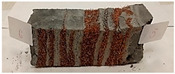
12F-H_R8	32.792; 8.986; 25.730; 7.141; 14.310; 11.536	15.141	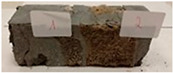 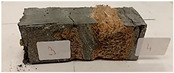 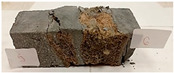

**Table 8:** Compressive strength of eco-friendly layered geopolymer composites (three bars).

Three Short Bars			
ID	R_c_ [MPa]	Avg. R_c_ [MPa]	Photo
RG referencyjna	48.847; 37.496; 63.791; 58.665; 25.592; 37.986	45.749	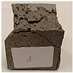 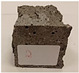 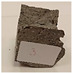 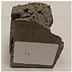 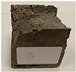 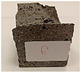
4F-H_R4	5.015; 5.019; 24.464; 6.626; 32.944; 31.043	16.788	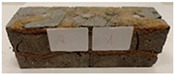 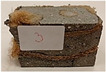 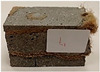 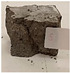 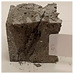
4C_R4	3.347; 2.792; 5.204; 4.352; 23.167; 30.014	9.018	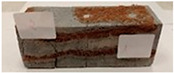 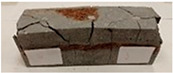 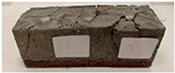
4F-H_R8	13.690; 10.506; 7.275; 6.161; 28.517; 25.532	14.251	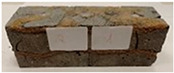 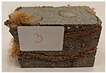 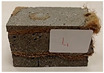 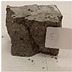 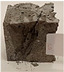
4C_R8	9.237; 9.567; 15.215; 14.164; 13.895; 12.305	12.483	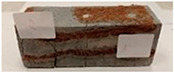 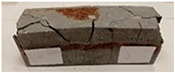 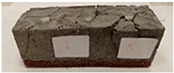
8F-H_R4	4.627; 5.335; 4.384; 3.583; 2.669; 2.386	3.816	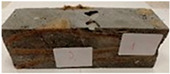 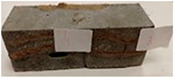 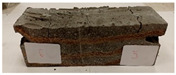
8C_R4	3.507; 4.243; 3.949; 2.140; 4.312; 3.674	3.843	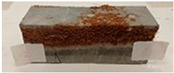 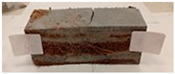 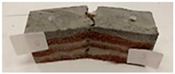
10C_R8	2.763; 11.014; 16.480; 13.012; 7.851; 12.801	11.170	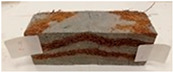 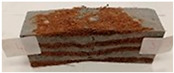 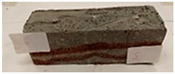
12F-H_R8	14.580; 27.444; 9.356; 14.066; 4.149; 5.509	10.878	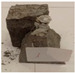 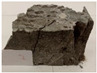 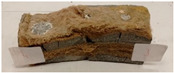 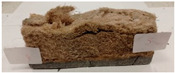

**Table 9:** Thermal conductivity of eco-friendly layered geopolymer composites.

ID	3 Measurements	Mean λ at 0–20 °C	Standard Deviation	Decrease in λ
	[W/m·K]	[W/m·K]	[W/m·K]	[%]
RG	1.24844; 1.04082; 1.08517	1.12481	0.08927	-
4F-H_R4	1.10744; 1.17157; 1.08438	1.12113	0.03689	↓0.3
4C_R4	0.99458; 0.79024; 0.82668	0.87050	0.08899	↓23
4F-H_R8	0.90878; 0.99740; 1.14021	1.01546	0.09534	↓10
4C_R8	0.98668; 1.00996; 0.95804	0.98489	0.02123	↓12
8F-H_R4	1.02380; 0.80770; 0.91145	0.91431	0.08825	↓19
8C_R4	0.73174; 0.50054; 0.71228	0.64819	0.10470	↓42
10C_R8	0.48413; 0.32585; 0.39058	0.40019	0.06497	↓64
12F-H_R8	0.41672; 0.44868; 0.57366	0.47969	0.06772	↓57

## Data Availability

The original contributions presented in the study are included in the article, further inquiries can be directed to the corresponding author.
